# Chylothorax after surgery on congenital heart disease in newborns and infants -risk factors and efficacy of MCT-diet

**DOI:** 10.1186/1749-8090-5-127

**Published:** 2010-12-13

**Authors:** Eva S Biewer, Christoph Zürn, Raoul Arnold, Martin Glöckler, Jürgen Schulte-Mönting, Christian Schlensak, Sven Dittrich

**Affiliations:** 1 Department of Congenital Heart Disease, University of Freiburg, Mathildenstraße 1, 79106 Freiburg, Germany; 2 Institute for Epidemiology and Biometrics, University of Freiburg, Stefan-Meier-Straße 26 79106 Freiburg, Germany; 3 Department for Cardiovascular Surgery, University of Freiburg, Hugstetter Straße 55, 79106 Freiburg, Germany; 4 Department of Pediatric Cardiology, University of Erlangen-Nuernberg, Erlangen, Loschgestraße 15, 91054 Erlangen, Germany

## Abstract

**Objectives:**

To analyze risk factors for chylothorax in infants after congenital heart surgery and the efficacy of median chain triglyceride diet (MCT). To develop our therapeutic pathway for the management of chylothorax.

**Patients and methods:**

Retrospective review of the institutional surgical database and patient charts including detailed perioperative informations between 1/2000 and 10/2006. Data analyzing with an elimination regression analysis.

**Results:**

Twenty six out of 282 patients had chylothorax (=9.2%). Secondary chest closure, low body weight, small size, longer cardiopulmonary bypass (242 ± 30 versus 129 ± 5 min) and x-clamp times (111 ± 15 versus 62 ± 3 min) were significantly associated with chylothorax (p < 0.05). One patient was cured with total parenteral nutrition (TPN) and one without any treatment. 24 patients received MCT-diet alone, which was successful in 17 patients within 10 days. After conversion to regular alimentation within one week only one chylothorax relapsed. Out of 7 patients primarily not responsive to MCT-diet, 2 were successfully treated by lysis of a caval vein thrombosis, 2 by TPN + pleurodesis + supradiaphragmatic thoracic duct ligation, one by octreotide treatment, and two patients finally died.

**Conclusions:**

Chylothorax may appear due to injury of the thoracic duct, due to venous or lymphatic congestion, central vein thrombosis, or diffuse injury of mediastinal lymphatic tissue in association with secondary chest closure. Application of MCT alone was effective in 71%, and more invasive treatments like TPN should not be used in primary routine. After resolution of chylothorax, MCT-diet can be converted to regular milk formula within one week and with very low risk of relapse.

## Introduction

Chylothorax is a frequent and serious complication associated with congenital heart surgery, which occurs with an incidence between 0.5% to 6.5%. It may be caused either by injury of the thoracic duct, increased pressure in the systemic veins exceeding that in the thoracic duct, or a central vein thrombosis [1-4]. The diagnosis is based on the milky or opalescent appearance of the fluid from the pleural spaces with high levels of triglycerides (> 110 mg/dl), proteins (> 20 g/L), and lymphocytes (> 80% of cells) [3]. These large losses of nutrients and immune cells put patients at risk of malnutrition, impair their immune system and may also lead to respiratory problems with the need of a pleural drain [5]. Published treatment strategies which aim to decrease or stop the lymphatic lymph flow are: long chain fatty acid free, median chain triglyceride (MCT)-enriched diet [2,6,7], total parenteral nutrition (TPN) [6,7], octreotide therapy [2,3,5,7]), optimization of hemodynamics (recanalisation of closed central veins), or closing the leakages by supraphrenic ligation or pleurodesis[8-10]. We reviewed our institutional database on congenital heart disease in a high risk population of newborns and infants for possible reasons of chylothorax and developed an algorithm for the therapeutic approach.

## Patients and methods

We carried out a systematic retrospective review of our institutional database on all surgeries of congenital heart disease in children within their first year of life at the Freiburg University Hospital between January 2000 and October 2006. Chylothorax was defined as the presence of significant pleural drainage losses with typical clinical appearance after the 5th day post op. Regularly we started milk feeding via the stomach tube as early as possible, regularly at day 3 postoperative. Therefore the typical white appearance of chylothorax was clearly observable at day 5 postoperative.

### Risk factors for chylothorax

Table 1 lists the potential risk factors which have been analyzed in our database. Additionally we assessed the duration of drainage, the day of maximum loss of chylous and the type and duration of treatment (fatty acid-free MCT-enriched diet, TPN, octreotide, recanalisation of thrombosed veins by lysis, supraphrenic ligation, surgical pleurodesis) as well as the procedure after successful treatment of chylothorax.

**Table 1:** Variables used for regression analysis

Anamnestical parameters	age, gender, weight, height	
**Parameters during operation**	CPB duration	If yes, perfusions-time, x-clamp-time, rectal temperature

**postoperatively**	The duration of sedation*, relaxation*, peritoneal dialysis, secondary chest closure	

**Other parameters**	Pre-operated, re-operation, death	

* The number of days of continuous iv-application were analyzed, for results see also table 2

### Statistics

Group comparison was performed with the Mann-Whitney-Test (SPSS program Version 15). A p-value < 0.05 was considered to be statistically significant. The parameters (listed in Table 1) were put in a multivariate binary logistic regression analysis with backward elimination (PROC logistic, SAS Version 9). In children who received repeated surgery in the first year of life only the data of the last operation was used for investigation.

### Treatment of chylothorax

On institutional consent, most patients with chylothorax were treated primarily with long chain fatty acid-free diet enriched with 1-2% MCT for at least 10 days. Additional treatment strategies were applied following clinical decision.

## Results

We analyzed the data of 282 neonatal or infant operations on congenital heart disease (between January 2000 and October 2006) 26 out of 282 patients (=9.2%) were diagnosed with chylothorax. The median duration of the chylous pleural effusions was 9 days (ranging from 3 to 59 days). The daily volume of chyle was 43 (18-183 ml/kg, [median, min-max], Table 2). In most cases, chylothorax was diagnosed after the correction of the transposition of great arteries (TGA), atrioventricular septal defect (AVSD) and after Norwood-I procedure in hypolastic left heart syndrome (HLHS, Table 3). The results of the multivariate regression analysis show that secondary chest closure (p < 0.0012 [1.8; 11.7]), long CPB-time (p = 0.0077 [2.2; 157.1]), postoperative sedation (p = 0.0017 [1.8; 11.7]) and reintubation (p = 0.001 [2.9; 24.9]) are associated with chylothorax. Duration of cardiopulmonary bypass (242 ± 30 min versus 129 ± 5 min) and the x-clamp-time (111 ± 15 min versus 62 ± 2.7 min) were longer in patients who subsequently developed chylothorax, p < 0.05 (Table 1). Patients with chylothorax had comparatively lower weight (median 3.9 ± 0.28 kg versus 4.8 ± 0.01 kg) and were of smaller body size (median 55.1 ± 1.4 cm versus 58.3 ± 0.5 cm), p < 0.05 (Table 4).

**Table 2:** Characteristics of patients with chylothorax

	median (range)
Duration of drainage (days)	9 (3- 59)

Duration of sedation (days)	2 (0-20)

Duration of relaxation (days)	0 (0-4)

Max. loss of chyle within 24 hours (ml/kg)	43 (18 - 183)

Day of max. loss (post-op day)	8 (5-52*)

Start of MCT-diet (post op-day), n = 24	9 (5-52*)

Start of octreotide (post op-day); n = 3	10, 17, 20

Duration of TPN treatment (days); n = 4	6 (2 -54)

Lowest serum total protein (g/l)**, ***	39 (30-49)

Lowest serum antithrombin III (%)**	59 (32-85)

Lowest serum quick (%)**	75 (42-101)

Lowest serum immunoglobulin G (mg/dl)**	220 (64-346)

* One patient developed chylothorax the first time on post-op-day 51st

**Lab-analyses during chylous-loss

*** Significantly lower compared to day 5 post-op analyses [40 (33-50) g/l], p < 0.05

**Table 3:** Diagnosis of the patients with chylothorax

Diagnosis	Frequency in patients with Chylothorax	Frequency in patients without Chylothorax
HRHS	3 (8%)*	36***

HLHS	4 (11%)**	36****

AVSD	4 (16%)*****	25

VSD	1 (3%)	41

ASD	1	0

TAC	2 (33%)	6

TGA	7 (28%)	25

DORV	1 (13%)	8

TOF	1 (4%)	29

CoA	1 (5%)	20

ALVS	1	2

others		28

* chylothorax in HRHS in one patient occurred after a neonatal operation (reconstruction of the RVOT), in one patient after bidirectional Glenn procedureand in one patient after valvuloplasty at the age of five month. CVP after bidirectional Glenn operation was below 18 mm of mercury during ventilation.

** In HLHS two cases of chylothorax occurred during the Norwood I procedure, two cases of chylothorax occurred after the bidirectional Glenn anastomosis. CVP was regular (14 - 16 mmHg) after extubation in both patients.

*** 9 out of 36 patients were bidirectional Glenn operations

**** 12 out of 36 operations were bidirectional Glenn operations

***** 2 out of 4 patients with chylothorax after correction of AVSD were suffering on Down-syndrome; 6 out of 25 children without chylothorax after correction of AVSD were without Down syndrome.

Most patients developed chylothorax after the correction of a transposition the great vessels. After HLHS and AVSD operations the chylothorax was seen as well. Abbr.: HRHS = hypoplastic right heart syndrome, HLHS = hypoplastic left heart syndrome, AVSD = atrium ventricular septal defect, VSD = ventricular septal defect, ASD = atrium septal defect, TAC = truncus arterious communis, TGA = transposition of the great vessels, DORV = double outlet right ventricle, TOF = Tetralogy of Fallot, CoA = coarctation of the aorta; ALVS = aorto left ventricular shunt

**Table 4:** Patient characteristics and operation variables

	no chylothorax	chylothorax	p
Number of patients (%)	256 (92.5%)	26 (7.5%)	

Age (days)	111 ± 5.8	82.4 ± 18.9	n. s.

Weight (kg)	4.8 ± 0.01	3.9 ± 0.28	< 0.05

Height (cm)	58.3 ± 0.5	55.1 ± 1.4	< 0.05

male: female	56 (%): 44 (%)	50 (%): 50 (%)	n. s.

Operations on CPB	83 (%)	85 (%)	n. s.

CPB duration (min)	129 ± 4.7	242.6 ± 29.6	< 0.05

x-clamp time (min)	61.6 ± 2.7	110.6 ± 15.3	< 0.05

Temperature (°C)	30.6 ± 0.31	28.7 ± 1.04	n. s.

Chylothorax patient are smaller and lighter and have a longer CPB duration and x-clamp time than non-chylothorax patients. Abbr.: n.s = not significant

**Figure 1:**
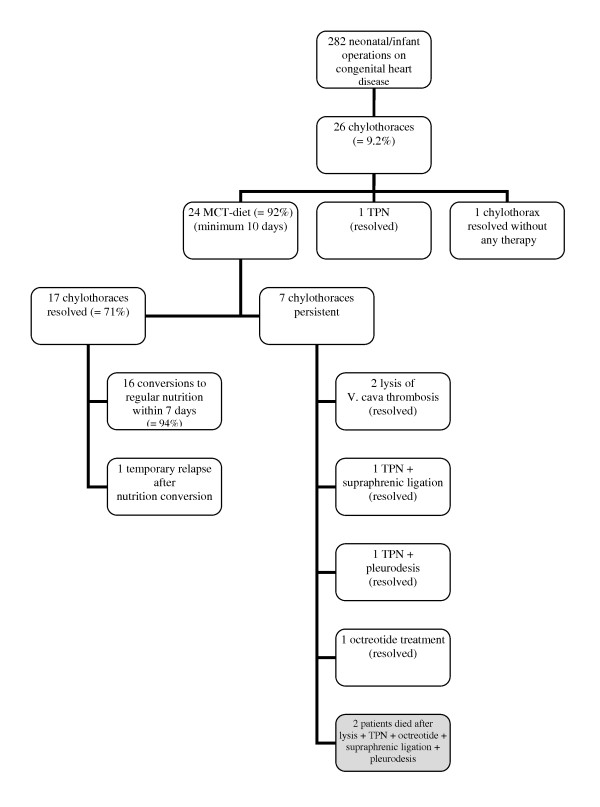
**Management of 26 patients with chylothoraces**. Chylothoraces were first-line treated with MCT-diet, which was effective in 71%. All patients but one tolerated rapid conversion to regular nutrition formula within 1 week after the chylothorax had disappeared. In 4 patients different additional treatments were effective. Two patients (grey setting box), both with low cardiac output and persistent capillary leackage died with remaining chylothorax despite numerous treatment attempts.

In 24 out of 26 patients with chylothorax, treatment was started with MCT diet. One patient was put on TPN (recovered from chylothorax), another recovered from chylothorax without any treatment. MCT-diet was successful in 17 out of 24 cases (=71%) and pleural drainages could be removed after a median of 9 days. After removal of the pleural drainages, gradual change of the diet to normal fatty nutrition was carried out successfully in 16 out of 17 patients (=94%) within one week (breast feeding in 10 cases). There was only one relapse of chylous effusion which required pleural drainage for one week (Figure 1).

MCT-diet alone was not successful in 7 out of 24 (=29%) patients. These 7 patients had much more daily drainage losses compared to patients successfully treated with MCT-diet (median loss 119 ml/kg/day versus 40 ml/kg/day). In 4 out of these 7 patients (=57%), a subclavian, innonimate or a superior caval vein thrombosis was diagnosed. A lysis therapy in 3 out of these 4 patients was successful in reopening the vein, but resolved chylothorax only in 2. In one patient who had a contraindication for a lysis therapy, chylothorax was successfully treated with TPN and supradiaphragmatic ligation of the thoracic duct. The 2 patients with remaining chylothorax suffered from severe capillary leakage and in one of these, the thrombosis appeared. The thrombosis was secondary to chylothorax, as it had been ruled out at the beginning of treatment. This suggests that the thrombosis resulted from the massive pleural drain losses and coagulation factor imbalances. Further attempts to treat chylous pleural and abdominal effusions including TPN, octreotide treatment, supraphrenic duct ligation and pleurodesis proved unsuccessful in both these patients. Both patients suffered from severe hemodynamic problems (elevated central venous pressures, low cardiac output) and died from intensive medicine complications on day 55 and 59 respectively (= 8% of all newborns/infants with chylothorax, = 29% of the patients unresponsive to MCT-diet).

## Discussion

Compared to the data found in the quoted literature[1-3], our study shows a high, 9% incidence of chylothorax in the high-risk newborn and infant population. Based on our study different reasons for chylothorax may exist, i.e. surgical damage of the thoracic duct, but also damage of minor chylous vessels, as well as lymphatic congestion due to elevated central venous pressure or central vein thrombosis[2,4,6]. Our observation that chylothorax is associated with secondary chest closure supports the thesis that non-specific mediastinal (lymphatic) tissue damage and postoperative impaired hemodynamics should be considered as important factors in the pathophysiology of chylothorax. Chylothorax was present in 28% of the arterial switch operations (7 out of 25 patients). In contrast, chylothorax occurred only in three patients with univentricular heart after bidirectional Glenn procedure, all without evidence for highly elevated CVP. This data suggest that elevated CVP is not the major driver for chylothorax in our series.

Our study shows that 10-day treatment of chylothorax using a long chain fatty acid free MCT-enriched diet was effective in 71% of affected patients. Moreover, 90% of recovered patients tolerated well the rapid conversion [1] to regular alimentation (mostly breast feeding) within one week after recovery, without relapse of chylothorax (Figure 1). This is especially important for newborns and infants as the growing brain is strongly dependent on the supply of balanced fatty acid nutrition [11] and breast feeding encourages mother-child interaction and neurological development [12]. Therefore we would not recommend a prophylactic administration of MCT diet after complex heart surgery without evidence of chylothorax. In contrast, TPN has much more side effects and has no higher efficacy in treating chylothorax [12]. Panthongviriyakul and Bines suggest using TPN in cases with elevated central venous pressures >15 mmHg [1]. When deciding over the use of TPN, its possible side-effects should be considered, such as an increased risk of nosocomial infections [13,14]. Patients with TPN are hungry and distressed, requiring sedatives that may have a detrimental effect on blood pressure and hemodynamics, and prolong the weaning of the respirator. Patients on TPN are dependent on central venous lines, through which they receive high osmolar fluids that ensure an adequate supply of calories. This puts them at risk of developing central vein thrombosis [15]. To avoid central lines, an oral nutrition with a low fat formula (basic-f) and intravenous application of lipids may be practical. Since central vein thrombosis has been identified as a possible cause of chylothorax, lysis treatment or an interventional procedure should be considered if a central vein thrombosis is diagnosed [16,17]. The limitations of our study are the restricted number of patients, heterogeneous diagnoses and operations, and the retrospective design. Due to these limitations, we could not methodically analyze the impact of the loss of pro- and anti-coagulation factors, total protein and immunoglobulins on infections, thromboses and on the outcome (Table 2).

**Figure 2:**
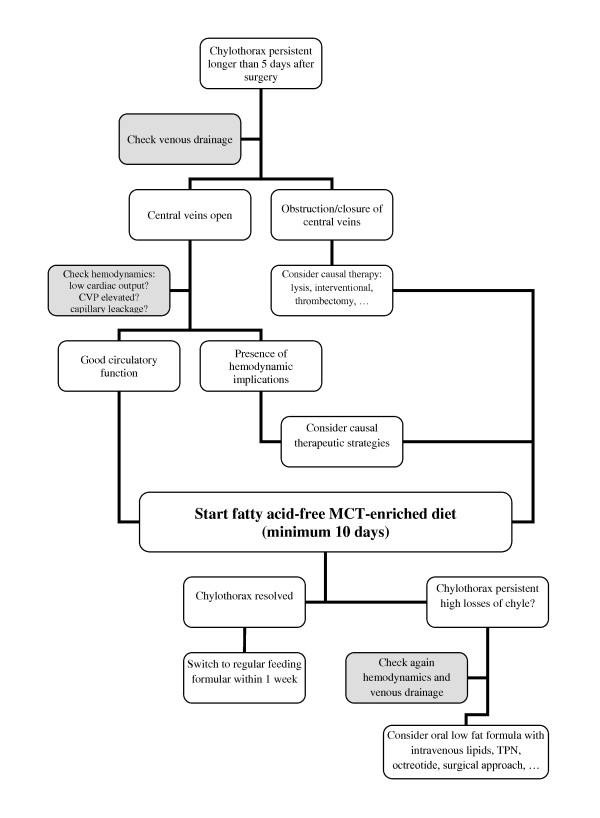
**Recommendation for the management of chylothorax**. Possibly treatable causes for chylothorax should be ruled out or treated before symptomatic therapy is started. Before more invasive and long-lasting therapies are started, one attempt to treat chylothorax with MCT-diet should be done (efficacy to treat chylothorax is 70% within acceptable treatment period). Abbr.: MCT = fatty acid-free MCT-enriched diet; TPN = total parenteral nutrition.

In our study, patients who did not respond to MCT-diet suffered higher chylous pleural losses and carried a limited prognosis of viability, as the reasons for chyle separation were not resolved. We therefore recommend an enlarged diagnostic work-up in cases where the patient's condition does not improve after ten days of MCT-diet (ruling out secondary central vein thrombosis, optimizing hemodynamics). Especially venous obstruction might be treated by transcatheter interventions (balloon angioplasty, mechanical thrombolysis or stenting) [18] or even by surgical thromectomie [16]. It is also important to consider early application of additional and more invasive treatment strategies like TPN, octreotide, supraphrenical ligation or pleurodesis in such cases (Figure 2).

In conclusion, we found that newborns and infants who have undergone complex cardiac surgery are at the highest risk for chylothorax. These patients' risk is further increased in cases with secondary chest closure. In the majority of the patients (71% in our study), chylous leakage was temporary and could be treated effectively using a long chain fatty acid-free MCT-enriched diet, suggesting that the general use of longer and more invasive treatment is not necessary. MCT-enriched diet has no considerable negative impact on the general state of health, which is of special importance for newborns and infants and their particular nutritional requirements. Patients with persistent chylothorax carry a limited prognosis, which means that application of additional treatment strategies including surgical options should be considered in time. In particular, central vein thrombosis should be treated energetically.

## Competing interests

The authors declare that they have no competing interests.

## Authors' contributions

ESB conceived the study, participated in literature search, drafted the manuscript. CZ participated in drafting the manuscript, had primary responsibility for data collection. RA participated in literature search and drafting the manuscript. JSM participated in the design of the study and performed the statistical analysis. MG participated in literature search and drafting, reviewed the manuscript. CS participated in its design and coordination. SD supervised the work, participated in drafting the manuscript, reviewed the manuscript.

All authors read and approved the final manuscript.
